# Supplementary material for: Mass Spectrometry-Based Workflow for the Identification and Quantification of Alternative and Canonical Proteins in Pancreatic Cancer Cells
Source: Cells. 2024 Nov 28;13(23):1966. doi: 10.3390/cells13231966 (PMC11640293; doi:10.3390/cells13231966)
Supplement: Supplementary file 1 [file cells-13-01966-s001.zip › Supplementary_Figures.pdf]

Supplementary Figure S1

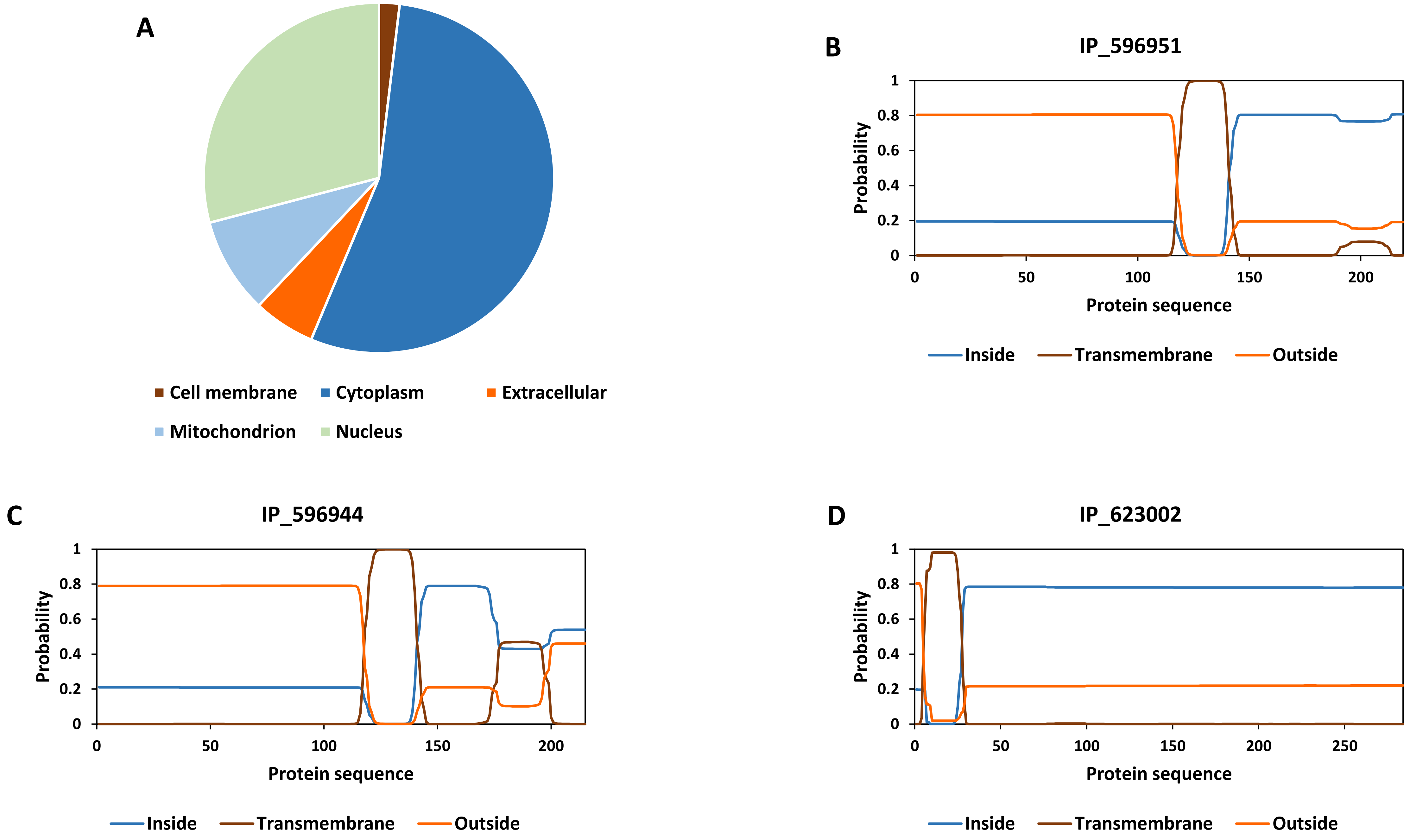

Supplementary Figure S2

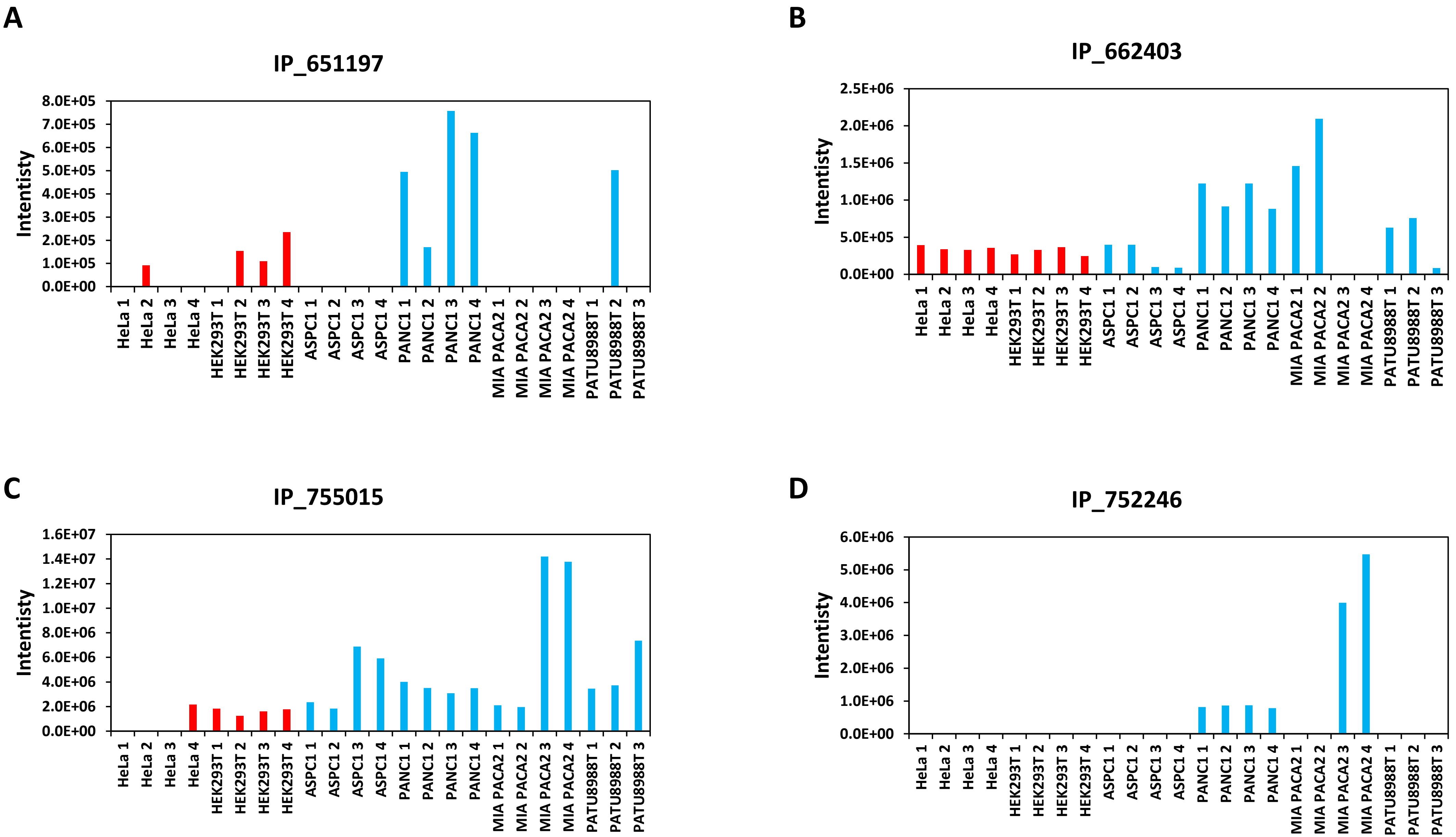

Supplementary Figure S3

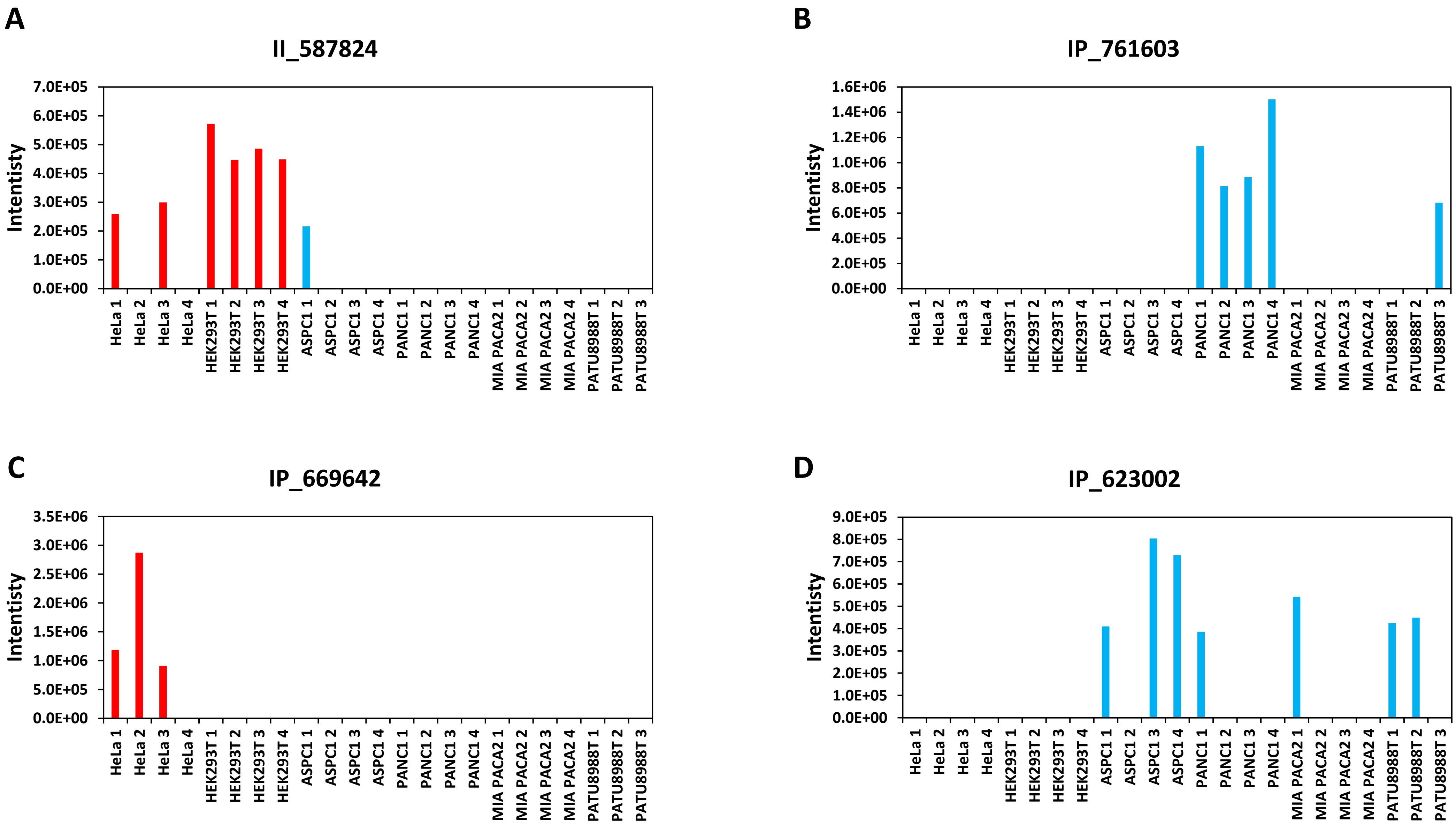

Supplementary Figure S4

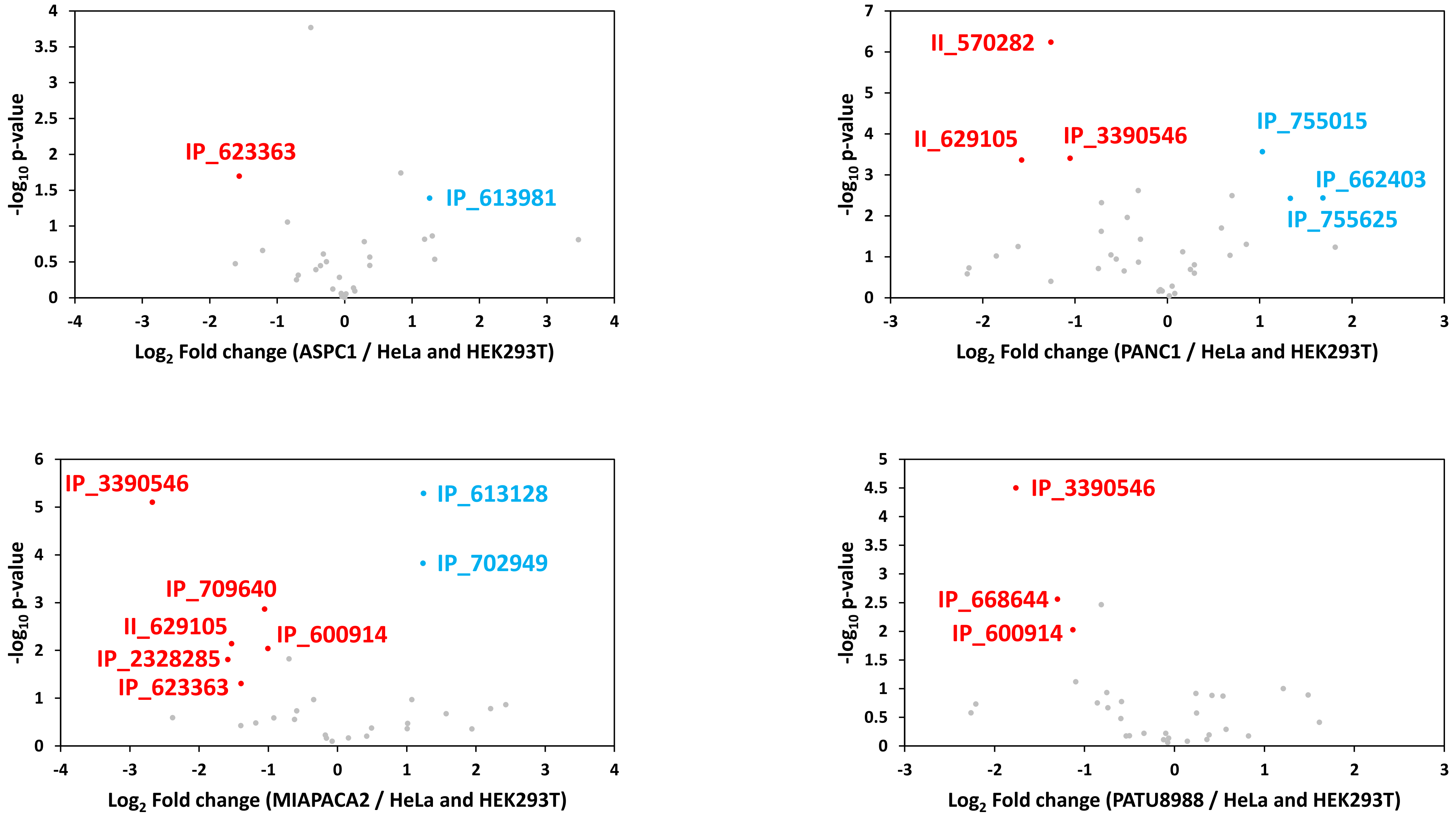

Supplementary Figure S5

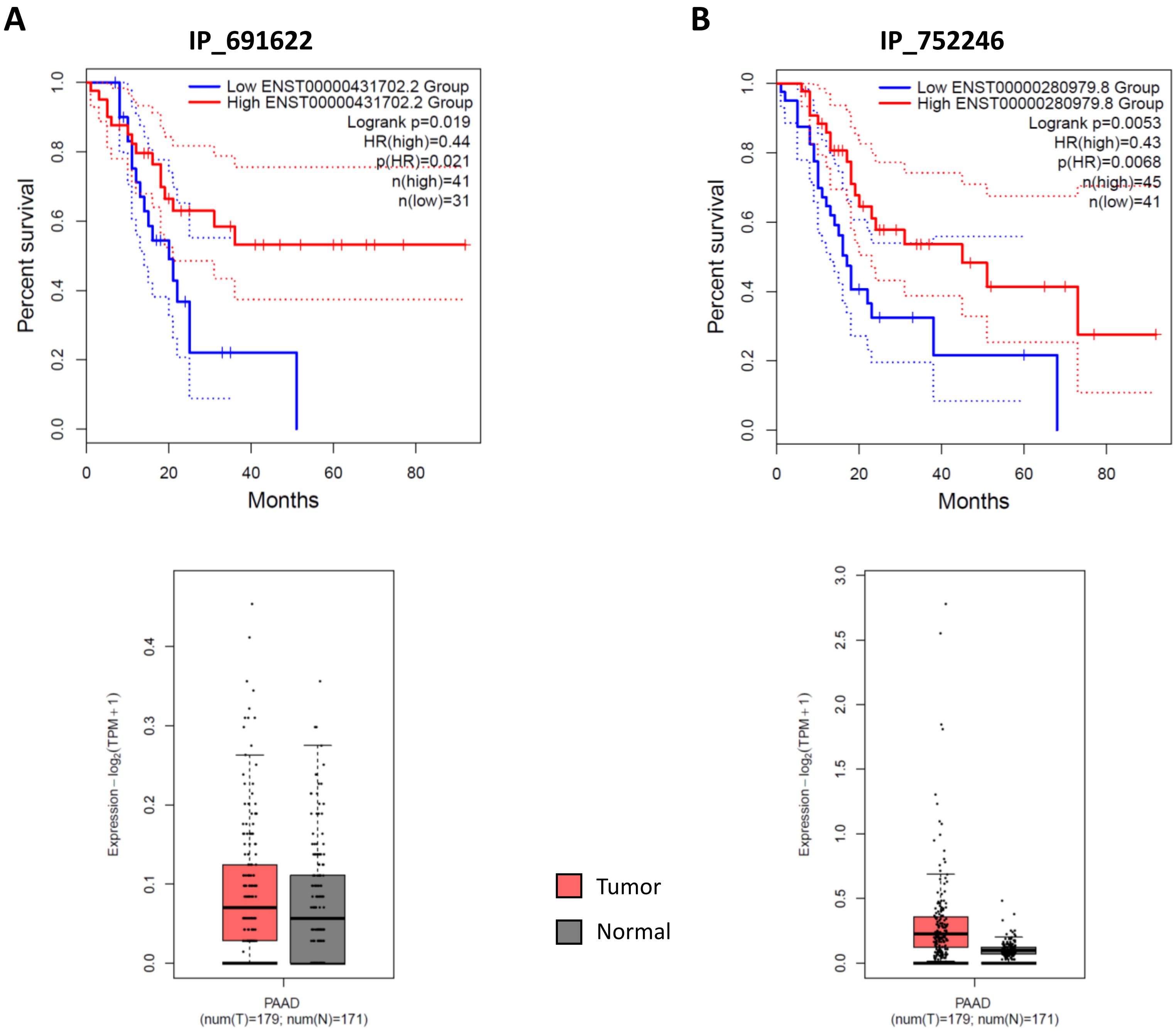

Supplementary Figure S6

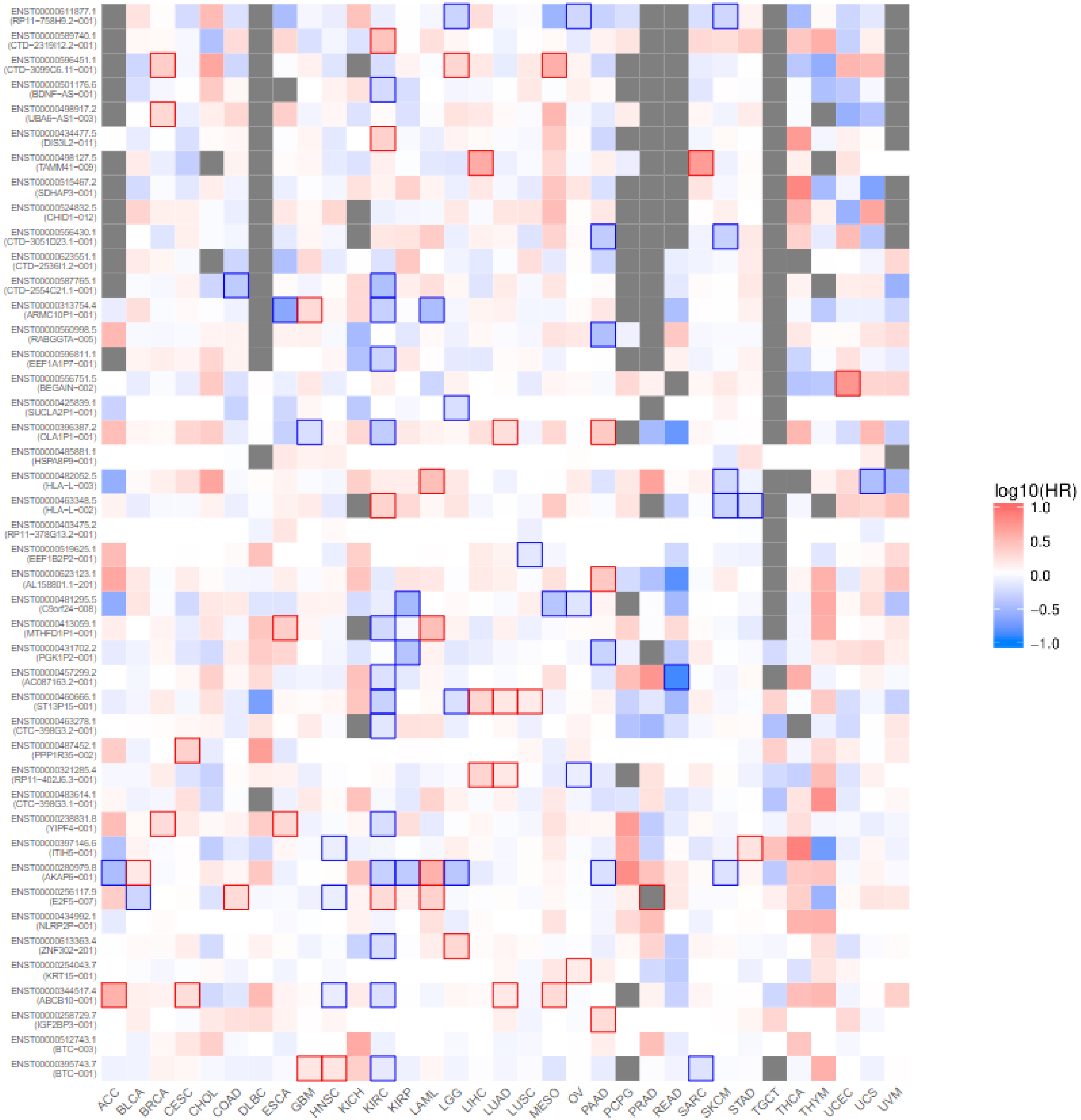

Supplementary Figure S7

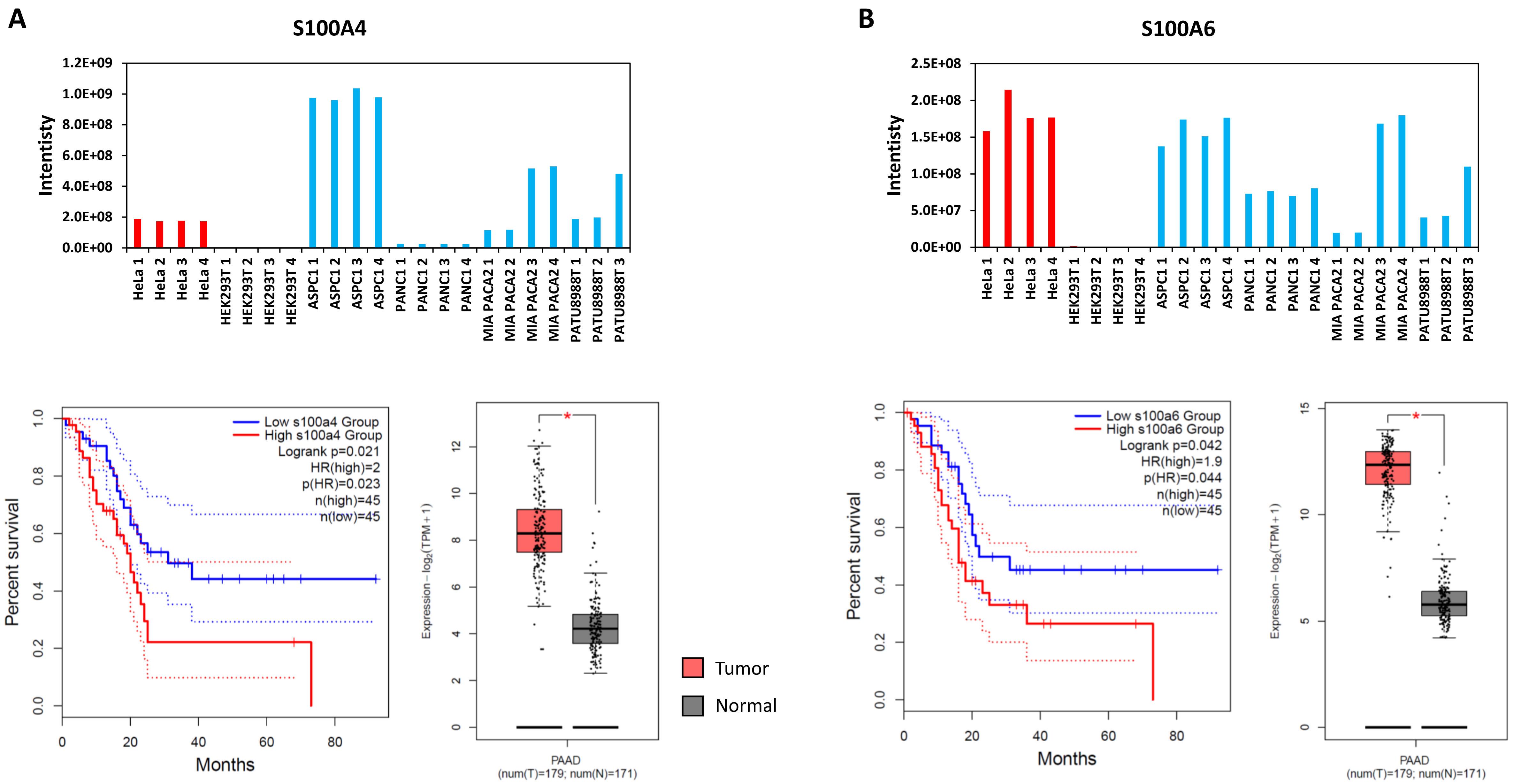

Figure S1: Prediction of AltProts subcellular localization using DeepLoc (A) and probability of the presence of transmembrane domain within the sequence of the Alt-Prot IP_596951 (B), IP_596944 (C) and IP_623002 (D) as predicted TMHMM - 2.0.

Figure S2: Chart displaying the protein intensity measured for the AltProts IP_651197 (A), IP_662403 (B), IP_755015 (C) and IP_752246 (D) across all the replicates of all the cell lines used in this study.

Figure S3: Chart displaying the protein intensity measured for the AltProts II_587824 (A), IP_761603 (B), IP_669642 (C) and IP_623002 (D) across all the replicates of all the cell lines used in this study.

Figure S4: Volcano plot representing the log 2 ratio for each pancreatic cell line pancreatic cell lines versus HeLa and HEK293T for each AltProt quantified in the DIA analysis and the corresponding Welch's t-test p-value (-Log10 transformed). The blue dots represent the AltProts more abundant in the different pancreatic cancer cell lines (p-value < 0.05 and Log2 fold change > 1), red dots represent the AltProts more abundant in HeLa and HEK293T cells (other cell lines) (p-value < 0.05 and Log2 fold change < 1), and gray dots represent AltProts not differentially expressed between the different pancreatic cancer cell lines and HeLa and HEK293T.

Figure S5: Survival and expression analysis of PDAC patients of mRNAs encoding the IP_691622 (A) and IP_752246 (B) AltProts.

Figure S6: Survival analysis for all the RNAs encoding AltProts (all RNAs with data available in GEPIA2) in all the cancer types. Highlighted scares represent significant differences of survival between high and low expression groups.

Figure S7: Chart displaying the protein intensity measured for the Proteins S100A4 (A) and S100A6 (B), as well as survival and expression analysis of PDAC patients for these genes.
